# *Matrix metalloproteinase* gene polymorphisms and periodontitis susceptibility: a meta-analysis involving 6,162 individuals

**DOI:** 10.1038/srep24812

**Published:** 2016-04-20

**Authors:** Hong Weng, Yan Yan, Ying-Hui Jin, Xiang-Yu Meng, Yuan-Yuan Mo, Xian-Tao Zeng

**Affiliations:** 1 Center for Evidence-Based and Translational Medicine, Zhongnan Hospital of Wuhan University, Wuhan 430071, P. R. China; 2 Department of Stomatology, Taihe Hospital, Hubei University of Medicine, Shiyan 442000, P. R. China; 3 Evidence-Based Nursing Center, School of Nursing, Tianjin University of Traditional Chinese Medicine, Tianjin 300193, P. R. China; 4 Department of Stomatology, Daping Hospital & Research Institute of Surgery, Third Military Medical University, Chongqing 400042, P. R. China

## Abstract

We aimed to systematically investigate the potential association of *matrix metalloproteinase* (*MMP*)-9, -3, -2, and -8 gene polymorphisms with susceptibility to periodontitis using meta-analysis. A literature search in PubMed, Embase, and Web of Sciencewas conducted to obtain relevant publications. Finally a total of 16 articles with 24 case-control studies (nine on *MMP*-9-1562 C/T, seven on *MMP*-3-1171 A5/A6, four on *MMP*-2-753C/T, and four on *MMP*-8-799 C/T) were considered in this meta-analysis. The results based on 2,724 periodontitis patients and 3,438 controls showed that *MMP*-9-1562C/T, *MMP*-3-1171 A5/A6, and *MMP*-8-799C/T polymorphisms were associated with periodontitis susceptibility. No significant association was found between *MMP*-2-753 C/T and periodontitis susceptibility. Subgroup analyses suggested that the *MMP*-9-1562 C/T polymorphism reduced chronic periodontitis susceptibility and *MMP*-3-1171 A5/A6polymorphism increased chronic periodontitis susceptibility. In summary, current evidence demonstrated that *MMP*-9-753 C/Tpolymorphism reduced the risk of periodontitis, *MMP*-3-1171 5A/6A and *MMP*-8-799 C/Tpolymorphisms increased the risk of periodontitis, and *MMP*-2-753 C/T was not associated with risk of periodontitis.

Periodontitis is considered as an inflammatory disease caused by bacterial infection and characterized by loss of connective tissue and alveolar bone^1^,^2^. It is classified into two major types, namely chronic periodontitis (CP) and aggressive periodontitis (AgP)^3^,^4^, and is generally regarded as one of the most common diseases around the world with a prevalence of 15–20%^5^. Of greater importance, periodontitis has been suggested to be associated with other disorders such as head and neck cancer, coronary heart disease, and chronic obstructive pulmonary disease^6^,^7^,^8^,^9^. Therefore, to elucidate the etiology, identify risk factors, and prevent its onset are very important. In the past few years, great efforts had been taken to elucidate potential background leading to the etiology of periodontitis. In addition to bacterial infection, individuals’ susceptibility to periodontitis is likely to be of major importance in determining the manifestation and progression of the disease^10^. Genetic factors, such as cyclooxygenase-2 gene polymorphisms and interleukin gene polymorphisms had been demonstrated by meta-analyses that whether they were associated with periodontitis susceptibility^11^,^12^,^13^,^14^,^15^,^16^.

Periodontal health needs a balance between tissue destruction enzymes, e.g. *matrix metalloproteinase* (MMP), and its inhibitors. The MMP is a family of proteolytic enzymes involved in matrix remodeling and basement membranes in the begging and developing course of a wide range of diseases^17^. In addition, it has been confirmed to be involved in the pathogenesis of periodontitis^18^. Many meta-analyses showed that *MMP*-1 1607 G1/G2 gene polymorphism was associated with CP susceptibility, as well with the severity of disease condition^19^,^20^. Regarding *MMP*-9-1562 C/T polymorphism, a meta-analysis indicated that it had no association with periodontitis^19^, but another meta-analysis suggested that it might be involved in the development of periodontitis^21^. Therefore, we determined to perform a meta-analysis with improved quality and to investigate the interaction association between gene polymorphism and environmental factors such as smoking. Moreover, many molecular epidemiological studies has been conducted to investigate the association between *MMP*-3-1171 A5/A6, *MMP*-8-799 C/T, and *MMP*-2-753 C/T polymorphisms and periodontitis susceptibility. Nevertheless, the results still remain inconsistency and individual studies based on small sample sizes have low statistical power to investigate the real association^22^. These facts warranted us to perform a meta-analysis to further investigate the role of these polymorphisms in the pathogenesis of periodontitis.

## Methods

This study followed the Preferred Reporting Items for Systematic Reviews and Meta-Analyses (PRISMA) statementin reporting this meta-analysis^23^.

### Eligible criteria

The following criteria were used for literature selection: 1) articles that explored the association between *MMP* gene polymorphisms and periodontitis (CP and/or AgP) susceptibility; 2) the study design was cohort or case-control study; 3) there are adequate data to estimate odds ratios (ORs) and corresponding 95% confidence intervals (CIs). Two investigators independently screened all papers by title or abstract and then by a full content evaluation. Any discrepancy between the two authors was solved by discussion with a third investigator.

### Search strategy

A comprehensive electronic database search was conducted for studies that examined associations between *MMP* polymorphisms and periodontitis. We utilized the PubMed, Embase, and Web of Science citation index to identify papers in which *MMP* polymorphisms were examined in patients with periodontitis and controls (up to 20 September, 2015). In addition, bibliographies of all potentially relevant studies and recently reviews were reviewed to identify additional articles not indexed by the aforementioned databases. The following text words and MeSH terms were searched: “*matrix metalloproteinase*” (MeSH term and text word), “MMP” (text word), “genetic, polymorphism” (MeSH term), “polymorphism” (text word), “periodontal disease” (MeSH term and text word), and “periodontitis” (MeSH and text word). We limited the search to studies that were carried out on humans and were written in English.

### Data extraction and quality assessment

Two authors independently extracted the data and assessed the methodological quality. The following data were extracted from each study: first author’s surname, publication year, country, ethnicity, type of disease, source of control, sample size, number of genotype distribution, Hardy-Weinberg equilibrium (HWE) for controls, site of polymorphism, genotyping method, smoking percentile, and other factors for environment. Quality assessment of included studies was evaluated using the Newcastle-Ottawa scale (NOS) by two authors (*P* = 0.90 for κ test) independently^22^.

### Data Analysis

A *χ*^*2*^test was used to assess the deviation of genotype distribution from HWE among controls. We performed meta-analyses using allelic contrast, homozygote contrast, heterozygote contrast, recessive, and dominant models. The associations between the polymorphisms and periodontitis risk were assessed by ORs and corresponding 95% CIs. Cochran’s *Q* metric was used to assess between tric was used to assess between-study heterogeneity. When the *Q* metric (*P* < 0.1) indicated significant heterogeneity among studies, the random-effects model (the Der-Simonian and Laird method) was applied to perform the meta-analysis. If the between study heterogeneity was not significant (*P* ≥ 0.1), the fixed-effects model (Mantel-Haenszel method) was used. We quantified the degree of heterogeneity using *I*^2^ statistic^24^. *I*^2^ ranges between 0 and 100%, and stands for the proportion of inter-study variability attributable to heterogeneity rather than random error. *I*^2^ values of 75%, 50%, and 25% were defined as high, moderate, and low estimates, respectively. If the number of included studies was applicable, we conducted stratified analyses on the bias of type of disease, ethnicity, agreement with HWE for controls, source of control, severity of CP, smoking status, and genotyping method. Funnel plots are used to detect publication bias. In addition, we performed Egger’s linear regression test to measure the asymmetry of funnel plot (*P* < 0.05)^25^. The meta-analysis was conducted using the Stata 12.0 software (Stata Corp LP, College Station, TX, USA), and the *P* value was two-sided.

## Results

### Study identification and characteristics

**Figure 1:**
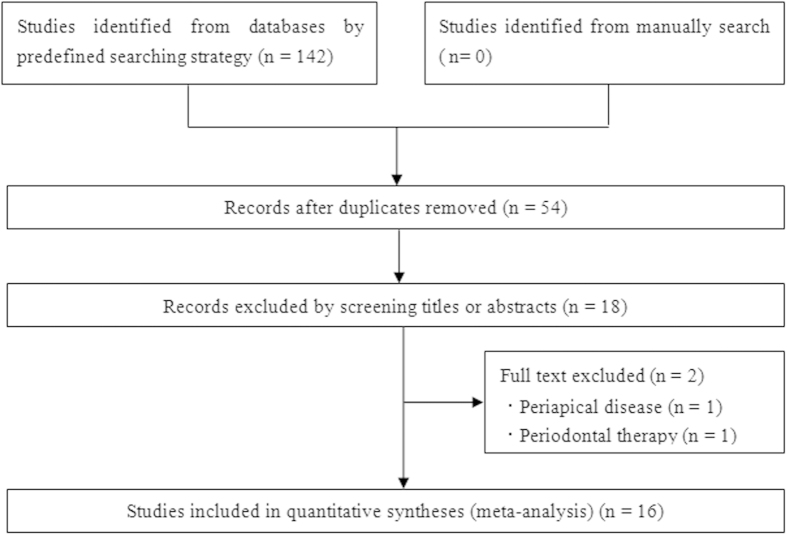
Flow chart for this meta-analysis.

As shown in Fig. 1, we initially identified 142 articles. Finally we included 16 articles^26^,^27^,^28^,^29^,^30^,^31^,^32^,^33^,^34^,^35^,^36^,^37^,^38^,^39^,^40^,^41^ with 24 case-control studies involving 2,724 cases and 3,438 controls. Among them, there were four polymorphisms of *MMP* gene included in our meta-analysis: nine on *MMP*-9-1562 C/T, seven on *MMP*-3-1171 A5/A6, four on *MMP*-2-753C/T, and four on *MMP*-8-799 C/T. Two of these studies^28^,^37^ contained data on two different groups (CP and AgP), which were considered independently. One article contained two independent case-control studies in different countries and in this study only allele number was available^39^. Three articles^27^,^40^,^41^ were focused on two polymorphisms, and two articles^31^,^32^ were focused on three polymorphisms, which were treated as independent case-control studies as well. Four articles with six case-control studies did not satisfy the HWE for control group^26^,^39^,^40^,^41^. Main characteristics of included studies were summarized in Table 1.

### MMP-9-1562 C/T polymorphism and periodontitis susceptibility

**Figure 2:**
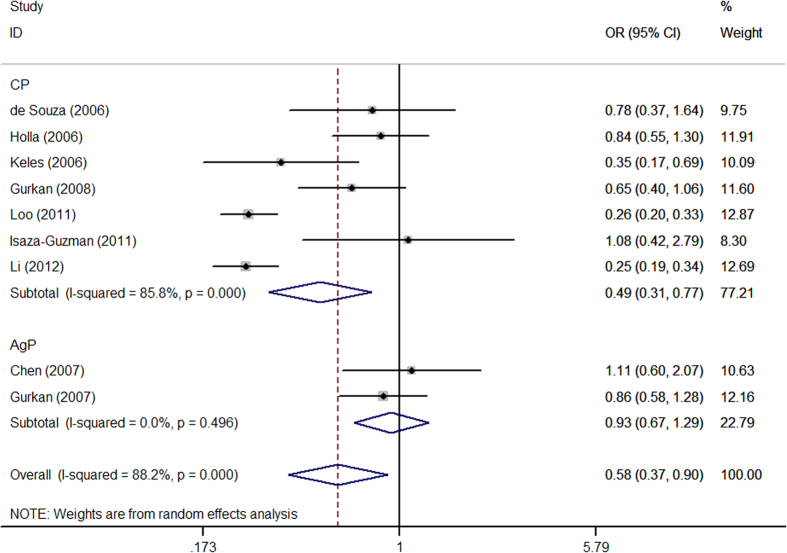
Forest plot for MMP-9-1562 C/T polymorphism associated with periodontitis susceptibility in C versus T allele comparison based on random-effects model.

In all study subjects, meta-analysis showed a reduced risk between the *MMP*-9-1562 C/T polymorphism and periodontitis susceptibility in all tested genetic model (T vs. C: OR = 0.58, 95% CI = 0.37-0.90; TT vs. CC: OR = 0.17, 95% CI = 0.13–0.23; CT vs. CC: OR = 0.61, 95% CI = 0.41–0.93; TT + CT vs. CC: OR = 0.54, 95% CI = 0.32–0.93; TT vs. CC + CT: OR = 0.28, 95% CI = 0.21–0.36) with some evidence of between-study heterogeneity (Table 2, Fig. 2). Sensitivity analysisby excluding studies with control inconsistent with HWE showed that the decreased risk was only observed in recessive model (OR = 0.41, 95% CI = 0.18–0.93). Stratification analysis by type of disease indicated that individuals were more susceptible to CP than AgP (Table 2).

### MMP-3-1171 A5/A6 polymorphism and periodontitis susceptibility

Meta-analysis of the *MMP*-3-1171 A5/A6 polymorphism showed an elevated risk between the polymorphism and periodontitis susceptibility in three tested genetic model (A5 vs. A6: OR = 1.45, 95% CI = 1.26–1.66; A5/A5 vs. A6/A6: OR = 2.32, 95% CI = 1.42–3.81; A5/A5 vs. A6/A5 + A6/A6: OR = 2.03, 95% CI = 1.59–2.59) with low between-study heterogeneity (Table 3. Stratification by disease type indicated that individuals were more susceptible to CP rather than AgP (Table 3).

### MMP-2-753C/T and MMP-8-799 C/T polymorphisms and periodontitis susceptibility

Meta-analysis of the *MMP*-2-753 C/T showed no association between thepolymorphism and periodontitis susceptibility (T vs. C: OR = 1.13, 95% CI = 0.88–1.44; TT vs. CC: OR = 1.25, 95% CI = 0.58–2.73; CT vs. CC: OR = 1.14, 95% CI = 1.14, 95% CI = 0.85–1.53; CT + TT vs. CC: OR = 1.15, 95% CI = 0.87–1.53; TT vs. CC + CT: OR = 1.18, 95% CI = 0.55–2.56) with no between-study heterogeneity (*I*^2^ = 0% for all genetic models) (Table 4). The results of stratification analyses according to disease type, ethnicity, and smoking statuswere similar to the overall results (Table 4).

Meta-analysis of the *MMP*-8-799 C/T showed an increased risk between the polymorphism and periodontitis susceptibility in four genetic model (T vs. C: OR = 1.61, 95% CI = 1.11–2.35; TT vs. CC: OR = 2.26, 95% CI = 1.03–4.97; CT vs. CC: OR = 2.18, 95% CI = 1.19–4.00; CT + TT vs. CC: OR = 2.22, 95% CI = 1.21–4.08) with moderate to highbetween-study heterogeneity (Table 4). Subgroup analyses according to disease type, ethnicity, and smoking status showed that the increased risk was predominant in CP, Asians, and non-smokers (Table 4).

### Publication bias

Due to limitations of the quantity of included studies, we just test the publication bias for *MMP*-9-1562 C/T and *MMP*-3-1171 A5/A6 polymorphisms. The funnel plots based on allele model for *MMP*-9-1562 C/T and *MMP*-3-1171 A5/A6 polymorphism were asymmetry and indicated that publication bias probably existed in the present study. The Egger’s test showed there was some publication bias existed in the *MMP*-9-1562 C/T allele model (Table 2).

## Discussion

In the present meta-analysis, we aggregated data from published studies to estimate genetic associations between *MMP* gene, namely *MMP*-2-753 C/T, *MMP*-3-1171 A5/A6, *MMP*-8-799 C/T, and *MMP*-9-1562 C/T polymorphisms, and periodontitis susceptibility. Our results provided some evidence to support an elevated risk between periodontitis susceptibility and *MMP*-3-1171 A5 allele and *MMP*-8-799 T allele, and a reduced risk between periodontitis susceptibility and *MMP*-9-1562 T allele. But our study provided no evidence to support an association between *MMP*-2-753 C/T polymorphism and periodontitis. Appreciable differences were identified in the etiology characteristic between CP and AgP^42^, indicating that there might be different genetic mechanism between them. In stratified analysis by disease type, their association with susceptibility of CP rather than AgP was observed. For *MMP*-9-1562 C/T polymorphism, we did not observe any meaningful associations in stratified analysis by ethnicity, severity of CP, and smoking status. The results were similar to *MMP*-3-1171 A5/A6 and *MMP*-2-753 C/T polymorphisms for periodontitis susceptibility. However, in stratified analysis by ethnicity and smoking status for *MMP*-8-799 C/T polymorphism indicated an elevated risk in Asian populations rather than Caucasian populations, and non-smokers rather than smokers.

To our knowledge, this is the first quantitative analysis that assessed the association between *MMP*-2-753 C/T, *MMP*-3-1171 A5/A6, and *MMP*-8-799 C/T polymorphisms and periodontitis susceptibility. With regard to *MMP*-9-1562 C/T polymorphism, two meta-analyses waspublished in 2013. *Pan et al.*^21^ indicated that *MMP*-9-1562 C/T polymorphism might be involved in the development of periodontitis. However, *Song et al.*^19^ suggested no association between *MMP*-9-1562 C/T polymorphism and periodontitis susceptibility. Compared with the previous meta-analyses, we had a lager sample size than them, which increased the statistical power, and we found a reduced risk of *MMP*-9-1562 T allele with periodontitis susceptibility. Genetic association researches designed to investigate relations between gene polymorphisms and complex outcomes must be interpreted with caution, because many factors could potentially affect the results. Therefore, we assessed the association with severity of CP and smoking status even though we did not observe any significant difference among the moderate and severe CP and no association among non-smokers. This might be caused by small sample size that only two studies^29^,^33^ presented the association between polymorphism and disease severity, two studies^31^,^32^ investigate the interaction between polymorphism and smoking status.

However, the present meta-analysis also has certain limitations that affect the interpretation of the results. First, the heterogeneity for *MMP*-9-1562 C/T polymorphism was high. Subgroup analyses suggested that the heterogeneity might come from the deviation of HWE, ethnicity, and genotyping method. Certainly, other clinical heterogeneity also might contribute to it, for instance different classification and diagnosis of periodontitis and differences on the oral examination by different clinicians. In addition, it is widely acknowledged that meta-analysis is a secondary analysis and we could not handle the problem of clinical heterogeneity in a meta-analysis. Therefore, we recommend that further studies should be designed as multi-center studies and utilizea unified criterion of disease. Second, although a comprehensive literature search was performed, it was likely that some publications were overlooked because of our language restriction for the literature search. In addition, the number of included studies for each polymorphism was limited. Therefore, the statistic power of present meta-analysis might be affected and the present results might be led to false positive or false negative rate. Third, we used genotype distributions and crude estimates of effect rather than adjusted estimates of association between polymorphism and periodontitis. Even though we investigated the interaction between polymorphism and disease severity and smoking status, the statistic power was limited due to most studies did not present the relevant data. Moreover, age, sex, and gene-gene interactions could not be assessed in our study due to insufficient data. Finally, the publication bias was of concern that small studies with negative results tend not to be published.

In conclusion, this meta-analysis with published data suggested that the *MMP*-2-753 C/T, *MMP*-3-1171 A5/A6, and *MMP*-9-1562 C/T polymorphisms were associated with periodontitis susceptibility and there is lack of association between the *MMP*-8-799 C/T polymorphism and periodontitis. Further studies with large sample size, gene-gene, and gene-environment detailed information are needed to validate the present results.

## Additional Information

**How to cite this article**: Weng, H. *et al.* *Matrix metalloproteinase* gene polymorphisms and periodontitis susceptibility: a meta-analysis involving 6,162 individuals. *Sci. Rep.* **6**, 24812; doi: 10.1038/srep24812 (2016).

## Figures and Tables

**Table 1:** Characteristics of the studies included in the meta-analysis.

Polymorphism	Reference	Year	Country	Ethnicity	Disease type	Control source	Case			Control			Smoking (%)		Genotyping method	*P*^*#*^ value	NOS score
							CC	CT	TT	CC	CT	TT	Case	Control			
MMP-9-1562 C/T	de Souza	2005	Brazil	Mixed	CP	HB	42	20	0	24	13	1	0	0	PCR-RFLP	0.62	7
	Holla	2006	Czech	Caucasian	CP	HB	122	43	4	93	37	5	26.0	29.6	PCR	0.59	8
	Keles	2006	Turkish	Caucasian	CP	HB	57	13	0	42	24	4	NA	NA	PCR	0.82	7
	Chen	2007	China	Asian	AgP	PB	62	15	2	101	26	1	NA	NA	PCR-RFLP	0.63	7
	Gurkan	2007	Turkish	Caucasian	AgP	HB	58	53	1	78	72	7	32.6	5.1	PCR	0.06	7
	Gurkan	2008	Turkish	Caucasian	CP	PB	54	32	1	52	52	3	47.1	3.7	PCR	0.02	6
	Loo	2011	China	Asian	CP	PB	143	73	64	43	72	135	NA	NA	PCR-RFLP	<0.01	6
	Isaza-Guzman	2011	Colombia	Mixed	CP	HB	58	11	0	47	6	1	14.6	4.9	PCR-RFLP	0.16	7
	Li	2012	China	Asian	CP	PB	68	26	28	99	156	277	93.4	97.7	PCR-RFLP	<0.01	6
MMP-3-1171 A5/A6							A5/A5	A5/A6	A6/A6	A5/A5	A5/A6	A6/A6					
	Itagaki	2004	Japan	Asian	AgP	HB	0	17	20	4	38	100	NA	NA	Taqman	0.87	8
	Itagaki	2004	Japan	Asian	CP	HB	5	58	142	4	38	100	NA	NA	Taqman	0.87	8
	Astolfi	2006	Brazil	Mixed	CP	PB	19	52	19	8	70	25	NA	NA	PCR-RFLP	<0.01	6
	Loo	2011	China	Asian	CP	PB	154	115	11	100	135	15	NA	NA	PCR-RFLP	<0.01	6
	Li	2012	China	Asian	CP	PB	75	44	3	213	283	36	93.4	97.7	PCR-RFLP	<0.01	6
	Letra*	2012	Brazil	Mixed	CP	HB	NA	NA	NA	121	114	64	NA	NA	Taqman	<0.01	6
	Letra*	2012	US	Caucasian	CP	HB	NA	NA	NA	51	91	62	NA	NA	Taqman	0.13	7
MMP-8-799C/T							CC	CT	TT	CC	CT	TT					
	Chou	2011	China	Asian	CP	PB	122	191	48	53	40	13	25.5	15.1	PCR-RFLP	0.22	8
	Chou	2011	China	Asian	AgP	PB	34	50	12	53	40	13	25	15.1	PCR-RFLP	0.22	7
	Holla	2012	Czech	Caucasian	CP	HB	88	163	90	84	134	60	30	28	PCR-RFLP	0.63	8
	Emingil	2014	Turkey	Caucasian	AgP	PB	11	57	32	76	66	25	29	3.6	TaqMan	0.10	8
MMP-2-753C/T							CC	CT	TT	CC	CT	TT					
	Holla	2005	Czech	Caucasian	CP	PB	107	38	4	93	30	4	NA	NA	PCR-RFLP	0.42	8
	Chen	2007	China	Asian	AgP	PB	63	15	1	98	28	2	NA	NA	PCR-RFLP	1.00	7
	Gurkan	2007	Turkish	Caucasian	AgP	HB	49	39	4	98	54	5	32.6	5.1	PCR	0.06	7
	Gurkan	2008	Turkish	Caucasian	CP	PB	51	32	4	67	37	3	47.1	3.7	PCR	0.43	6

CP = chronic periodontitis, AgP = aggressive periodontitis, NA = not available, HB = hospital-based, PB = population-based.

*P*^#^ for Hardy-Weinberg equilibrium, *Only allele number available in controls.

**Table 2:** Meta-analysis of the association between the MMP-9-1562 C/Tpolymorphism and periodontitis.

Genetic Model and Subgroup	Subgroups	N	Test of Association			Test of Heterogeneity			P* value
			OR	95% CI	*P* value	Model	*P* value	*I*^2^(%)	
T vs. C	Overall	9	0.58	0.37–0.90	0.015	R	<0.001	88.2	0.039
	HWE (yes)	6	0.8	0.64–1.00	0.052	F	0.198	31.6	
	AgP	2	0.93	0.67–1.29	0.656	F	0.496	0	
	CP	7	0.49	0.31–0.77	0.002	R	<0.001	85.8	
	Caucasian	4	0.72	0.57–0.90	0.005	F	0.122	48.2	
	Asian	3	0.39	0.20–0.73	0.004	R	<0.001	89.9	
	Mixed	2	0.89	0.49–1.59	0.684	F	0.596	0	
	HB	5	0.76	0.60–0.97	0.027	F	0.19	34.7	
	PB	4	0.44	0.25–0.79	0.006	R	<0.001	89.7	
	PCR	4	0.72	0.57–0.90	0.005	F	0.122	48.2	
	PCR-RFLP	5	0.52	0.29–0.93	0.029	R	<0.001	87.8	
	Moderate CP	2	0.65	0.40–1.07	0.09	F	0.358	0	
	Sever CP	2	0.98	0.64–1.50	0.926	F	0.343	0	
	Non-smokers	2	0.67	0.46–1.00	0.048	F	0.86	0	
TT vs. CC	Overall	9	0.17	0.13–0.23	<0.001	F	0.217	25.6	0.121
	HWE (yes)	6	0.4	0.18–0.89	0.026	F	0.405	1.7	
	AgP	2	0.74	0.05–12.16	0.836	R	0.082	66.8	
	CP	7	0.16	0.12–0.22	<0.001	F	0.564	0	
	Caucasian	4	0.3	0.12–0.76	0.011	F	0.578	0	
	Asian	3	0.18	0.09–0.38	<0.001	R	0.043	68.1	
	Mixed	2	0.23	0.02–2.25	0.206	F	0.883	0	
	HB	5	0.29	0.12–0.72	0.008	F	0.718	0	
	PB	4	0.19	0.10–0.36	<0.001	R	0.083	55	
	PCR	4	0.3	0.12–0.76	0.011	F	0.578	0	
	PCR-RFLP	5	0.16	0.11–0.22	<0.001	F	0.171	37.6	
	Moderate CP	2	0.34	0.06–2.08	0.245	F	0.831	0	
	Sever CP	2	0.75	0.20–2.81	0.667	F	0.563	0	
	Non-smokers	2	1.38	0.38–4.92	0.623	F	0.579	0	
CT vs. CC	Overall	9	0.61	0.41–0.93	0.02	R	<0.001	74.8	0.322
	HWE (yes)	6	0.87	0.66–1.14	0.293	F	0.406	1.6	
	AgP	2	0.97	0.65–1.46	0.896	F	0.906	0	
	CP	7	0.53	0.33–0.85	0.008	R	0.001	73.4	
	Caucasian	4	0.75	0.57–0.99	0.046	F	0.192	36.8	
	Asian	3	0.39	0.19–0.80	0.01	R	0.008	79.4	
	Mixed	2	1.09	0.56–2.11	0.808	F	0.453	0	
	HB	5	0.85	0.64–1.14	0.286	F	0.285	20.4	
	PB	4	0.43	0.25–0.76	0.004	R	0.007	75.3	
	PCR	4	0.75	0.57–0.99	0.046	F	0.192	36.8	
	PCR-RFLP	5	0.56	0.29–1.09	0.09	R	0.001	79.2	
	Moderate CP	2	0.7	0.39–1.24	0.218	F	0.319	0	
	Sever CP	2	1.06	0.64–1.74	0.667	F	0.414	0	
	Non-smokers	2	1	0.63–1.57	0.988	F	0.51	0	
TT + CT vs. CC	Overall	9	0.54	0.32–0.93	0.025	R	<0.001	86.9	0.104
	HWE (yes)	6	0.82	0.63–1.06	0.133	F	0.276	20.9	
	AgP	2	0.95	0.64–1.42	0.814	F	0.798	0	
	CP	7	0.46	0.26–0.82	0.009	R	<0.001	85.8	
	Caucasian	4	0.71	0.54–0.93	0.013	F	0.134	46.2	
	Asian	3	0.32	0.13–0.77	0.011	R	<0.001	89.9	
	Mixed	2	0.98	0.51–1.88	0.953	F	0.512	0	
	HB	5	0.79	0.59–1.04	0.098	F	0.21	31.7	
	PB	4	0.37	0.17–0.78	0.009	R	<0.001	88.8	
	PCR	4	0.71	0.54–0.93	0.013	F	0.134	46.2	
	PCR-RFLP	5	0.48	0.22–1.07	0.072	R	<0.001	88.6	
	Moderate CP	2	0.65	0.37–1.14	0.132	F	0.323	0	
	Sever CP	2	1.02	0.63–1.66	0.939	F	0.363	0	
	Non-smokers	2	1.02	0.66–1.58	0.935	F	0.439	0	
TT vs. CC + CT	Overall	9	0.28	0.21–0.36	<0.001	F	0.601	0	0.379
	HWE (yes)	6	0.41	0.18–0.93	0.033	F	0.437	0	
	AgP	2	0.75	0.05–12.36	0.372	R	0.08	67.3	
	CP	7	0.27	0.20–0.35	<0.001	F	0.897	0	
	Caucasian	4	0.33	0.13–0.83	0.018	F	0.624	0	
	Asian	3	0.27	0.21–0.36	<0.001	F	0.119	53	
	Mixed	2	0.23	0.02–2.22	0.202	F	0.915	0	
	HB	5	0.3	0.12–0.75	0.01	F	0.753	0	
	PB	4	0.28	0.21–0.37	<0.001	F	0.224	31.3	
	PCR	4	0.33	0.13–0.83	0.018	F	0.624	0	
	PCR-RFLP	5	0.27	0.21–0.36	<0.001	F	0.368	6.7	
	Moderate CP	2	0.37	0.06–2.26	0.284	F	0.891	0	
	Sever CP	2	0.73	0.20–2.72	0.64	F	0.618	0	
	Non-smokers	2	1.35	0.38–4.80	0.641	F	0.618	0	

N = number of studies, OR = odds ratio, CI = confidence interval, F = fixed model, R = random model, CP = chronic periodontitis, AgP = aggressive periodontitis, HWE = Hardy-Weinberg equilibrium, HB = hospital-based, PB = population-based.

P* value for publication bias (Egger’s test).

**Table 3:** Meta-analysis of the association between the MMP-3-1171 5A/6A polymorphism and periodontitis.

Genetic Model and Subgroup	Subgroups	N	Test of Association			Test of Heterogeneity			P* value
			OR	95% CI	*P* value	Model	*P* value	*I*^2^(%)	
A5 vs. A6	Overall	7	1.45	1.26–1.66	<0.001	F	0.263	21.8	0.721
	HWE (yes)	3	1.34	1.01–1.70	0.04	F	0.315	13.4	
	AgP	1	1.54	0.82–2.89	0.175	NA	NA	NA	
	CP	6	1.44	1.25–1.66	<0.001	F	0.178	34.4	
	Caucasian	1	1.55	1.05–2.30	0.029	NA	NA	NA	
	Asian	4	1.53	1.28–1.83	<0.001	F	0.135	46	
	Mixed^#^	2	1.25	0.96–1.62	0.095	F	0.477	0	
	HB	4	1.25	1.02–1.54	0.033	F	0.444	0	
	PB	3	1.62	1.35–1.95	<0.001	F	0.394	0	
	Taqman	4	1.25	1.02–1.54	0.033	F	0.444	0	
	PCR-RFLP	3	1.62	1.35–1.95	<0.001	F	0.394	0	
A5/A5 vs. A6/A6	Overall	5	2.32	1.42–3.81	0.001	F	0.371	6.2	0.412
	HWE (yes)	2	0.8	0.24–2.65	0.713	F	0.771	0	
	AgP	1	0.54	0.03–10.51	0.688	NA	NA	NA	
	CP	4	2.45	1.47–4.08	0.001	F	0.336	11.3	
	Asian	4	2.13	1.21–3.75	0.009	F	0.293	19.5	
	Mixed^#^	1	3.13	1.13–8.66	0.028	NA	NA	NA	
A6/ A 5 vs. A6/A6	Overall	5	1.26	0.92–1.71	0.148	F	0.458	0	0.367
	HWE (yes)	2	1.32	0.88–1.99	0.176	F	0.106	61.7	
	AgP	1	2.24	1.06–4.72	0.035	NA	NA	NA	
	CP	4	1.12	0.80–1.58	0.504	F	0.836	0	
	Asian	4	1.34	0.85–1.89	0.101	F	0.39	0.4	
	Mixed^#^	1	0.98	0.49–1.96	0.949	NA	NA	NA	
(A5/A5 + A6/A5) vs. A6/A6	Overall	5	1.94	0.92–4.13	0.083	R	<0.001	81.3	0.021
	HWE (yes)	2	1.65	0.30–8.98	0.565	R	<0.001	92.8	
	AgP	1	4.05	1.82–9.00	0.001	NA	NA	NA	
	CP	4	1.59	0.74–3.38	0.232	R	0.004	77.2	
	Asian	4	2.12	0.78–5.79	0.141	R	<0.001	85.9	
	Mixed^#^	1	1.56	0.78–3.10	0.207	NA	NA	NA	
A5/A5vs. (A6/A5 + A6/A6)	Overall	5	2.03	1.59–2.59	<0.001	F	0.323	14.4	0.458
	HWE (yes)	2	0.73	0.22–2.39	0.606	F	0.649	0	
	AgP	1	0.41	0.02–7.79	0.553	NA	NA	NA	
	CP	4	2.06	1.61–2.64	<0.001	F	0.317	14.9	
	Asian	4	1.95	1.51–2.52	<0.001	F	0.306	17	
	Mixed^#^	1	3.18	1.32–7.67	0.01	NA	NA	NA	

N = number of studies, OR = odds ratio, CI = confidence interval, F = fixed model, R = random model, CP = chronic periodontitis, AgP = aggressive periodontitis, HWE = Hardy-Weinberg equilibrium, HB = hospital-based, PB = population-based.

P* value for publication bias (Egger’s test), mixed^#^ for ethnicity.

**Table 4:** Meta-analysis of the association between the MMP-2-753C/T and MMP-8-799C/T polymorphisms and periodontitis.

Genetic Model and Subgroup	N	T vs. C		TT vs. CC		CT vs. CC		(CT + TT) vs. CC		TT vs. (CC + CT)	
		OR (95% CI)	*I*^2^ (%)	OR (95% CI)	*I*^2^ (%)	OR (95% CI)	*I*^2^ (%)	OR (95% CI)	*I*^2^ (%)	OR (95% CI)	*I*^2^ (%)
MMP-2-753 C/T											
Total	4	1.13 (0.88–1.44)	0	1.25 (0.58–2.73)	0	1.14 (0.85–1.53)	0	1.15 (0.87–1.53)	0	1.18 (0.55–2.56)	0
CP	2	1.11 (0.79–1.55)	0	1.20 (0.43–3.37)	0	1.12 (0.74–1.68)	0	1.12 (0.76–1.66)	0	1.16 (0.42–3.24)	0
AgP	2	1.15 (0.81–1.64)	29.9	1.33 (0.41–4.31)	0	1.18 (0.77–1.79)	32.8	1.18 (0.78–1.78)	39.3	1.21 (0.38–3.88)	0
Caucasian	3	1.19 (0.91–1.55)	0	1.33 (0.58–3.03)	0	1.23 (0.89–1.69)	0	1.23 (0.90–1.69)	0	1.24 (0.55–2.80)	0
Asian	1	0.84 (0.45–1.58)	NA	0.78 (0.07–8.76)	NA	0.83 (0.41–1.68)	NA	0.83 (0.42–1.64)	NA	0.81 (0.07–9.06)	NA
Non-smokers	3	1.27 (0.92–1.75)	0	1.38 (0.52–3.62)	0	1.35 (0.91–2.00)	0	1.35 (0.92–1.97)	0	1.24 (0.48–3.22)	0
Smokers	1	0.85 (0.36–1.99)	NA	1.10 (0.09–13.00)	NA	0.73 (0.26–2.08)	NA	0.77 (0.28–2.10)	NA	1.21 (0.10–14.00)	NA
MMP-8-799 C/T											
Total	4	1.61 (1.11–2.35)	81.6	2.26 (1.03–4.97)	80.8	2.18 (1.19–4.00)	81.5	2.22 (1.21–4.08)	94.5	1.44 (0.90–2.16)	47.3
CP	2	1.28 (1.07–1.54)	0	1.48 (1.02–2.15)	0	1.52 (0.86–2.69)	72	1.48 (1.13–1.95)	59.9	1.25 (0.90–1.73)	0
AgP	2	2.01 (0.98–4.11)	85.3	3.60 (0.61–21.33)	88.4	3.34 (1.11–10.04)	81.7	3.45 (0.95–12.55)	87.9	1.73 (0.68–4.42)	70.2
Caucasian	2	1.84 (0.78–4.32)	93.8	3.44 (0.58–20.47)	93.2	2.55 (0.51–12.76)	93.6	2.82 (0.53–14.96)	94.5	1.79 (0.89–3.61)	75
Asian	2	1.43 (1.11–1.85)	0	1.54 (0.89–2.67)	0	2.03 (1.40–2.93)	0	1.91 (1.35–2.70)	0	1.07 (0.64–1.79)	0
Non-smokers	4	1.79 (1.19–2.69)	79.4	2.88 (1.21–6.84)	78.3	2.37 (1.24–4.55)	77.2	2.52 (1.30–4.88)	80.5	1.64 (1.19–2.25)	38.7
Smokers	1	0.91 (0.58–1.42)	NA	0.81 (0.33–2.00)	NA	0.96 (0.46–1.99)	NA	0.91 (0.46–1.82)	NA	0.84 (0.39–1.81)	NA

OR = odds ratio, CI = confidence interval, CP = chronic periodontitis, AgP = aggressive periodontitis, NA = not available.
